# Acute and Subacute Toxicity Assessment of Andrographolide-2-hydroxypropyl-*β*-cyclodextrin Complex via Oral and Inhalation Route of Administration in Sprague-Dawley Rats

**DOI:** 10.1155/2022/6224107

**Published:** 2022-03-28

**Authors:** Shashi Chandrama Singh, Muskan Choudhary, Atul Mourya, Dharmendra Kumar Khatri, Pankaj Kumar Singh, Jitender Madan, Harshpal Singh

**Affiliations:** ^1^Research and Development Centre, Ambe Phytoextracts Private Limited, Pauri Garhwal, Uttarakhand, India; ^2^Department of Pharmaceutics, National Institute of Pharmaceutical Education and Research, Hyderabad, Telangana, India; ^3^Department of Biological Sciences, National Institute of Pharmaceutical Education and Research, Hyderabad, Telangana, India

## Abstract

**Objective:**

Acute and subacute toxicity analysis of AND-2-HyP-*β*-CYD complex was conducted in Sprague-Dawley (SD) rats following oral and inhalation routes of administration.

**Methods and Results:**

Single dose acute toxicity was carried out at 2000 mg/kg of AND-2-HyP-*β*-CYD complex, while the doses of 200, 400, and 666 mg/kg were administered, over a period of 28 days under repeated dose oral toxicity study. Hence, LD50 (lethal dose) was found to be >2000 mg/kg in addition to NOAEL (no observed adverse effect level) of 666 mg/kg. Correspondingly, single dose acute inhalation toxicity of AND-2-HyP-*β*-CYD complex was carried out at 5 mg/L/4 h/day and subacute inhalation toxicity at 0.5, 1, and 1.66 mg/L/4 h/day over a period of 28 days. The NOAEL and LOAEL (lowest observed adverse effect level) were estimated to be 0.5 mg/L/4 h/day and 1 mg/L/4 h/day, respectively.

**Conclusion:**

The findings of the present study would further be useful in assessing and utilizing the medicinal and therapeutic benefits of AND-2-HyP-*β*-CYD complex.

## 1. Introduction

Andrographolide (AND) is a diterpenoid with multiple biological activities, but most commonly employed for its anti-inflammatory action [1]. It is abundantly present in leaves and stems, followed by the seeds of plants belonging to *Andrographis* genus, commonly known as “Creat” or “Green Chiretta” [2]. The purified form of AND has been investigated for its anti-inflammatory effects in various stressful conditions, such as liver disorders, ischemia, arthritis, cancer, and oxidative stress [3–8]. Besides anti-inflammatory activity, AND also displays immunostimulatory action by efficaciously increasing CD4+ and CD8+ cells population [9]. All these properties of AND form the foundation for its clinical application against viral infections. Furthermore, these studies necessitate the development of a biopharmaceutically effective dosage form for oral and inhalation administration.

Previously, we have synthesized and characterized andrographolide-2-hydroxypropyl-*β*-cyclodextrin (AND-2-HyP-*β*-CYD) complex to augment the bioavailability of phytomolecules [10]. Thus, the evaluation of toxicity profile of AND-2-HP-*β*-CYD complex through oral and inhalation routes seems to be important and needs to be further studied.

Therapeutic entity mediated hepatotoxicity and nephrotoxicity are the foremost important reasons for the pharmaceutical withdrawals of promising chemical entities in clinical trials. In order to evaluate drug-induced hepatoxicity, alanine aminotransferase (ALT) biomarker plays the most important role followed by alkaline phosphate (ALP), albumin (ALB), and bilirubin (BIL). On the other hand, urea, phosphorous (PHOS), and serum creatinine (CREJ) levels are the commonly used end point indicators for the assessment of renal functions [11]. Therefore, evaluation of at least four serum parameters (hepatocellular and hepatobiliary serum biomarkers) has been recommended for toxicity profiling of therapeutically active compounds [12]. Hence, single dose acute (14 days) and repeated dose subacute (28 days) toxicity of AND-2-HyP-*β*-CYD complex was assessed following oral and inhalation routes of administration in Sprague-Dawley (SD) rats under a set of stringent in vivo parameters.

## 2. Materials and Methods

### 2.1. Ethics Statement

All animal studies were performed in accordance with the guidelines of Committee for the Purpose of Control and Supervision of Experiments on Animals (CPCSEA), Ministry of Fisheries, Animal Husbandry, and Dairying, Government of India, New Delhi. The study was approved by the Institutional Animal Ethics Committee (IAEC) vide protocol # NIP/PE/409 and # NIP/PC/392. The animals were examined and allowed to adapt the new environmental conditions for a week before the commencement of experiments.

### 2.2. Animals

Healthy male and female SD rats with average weight of 168.9 g were purchased from the certified suppliers. All the toxicity studies were conducted by strictly following Organization for Economic Cooperation and Development (OECD) guidelines. All the animals were housed separately in plastic cages according to their sex and maintained for 12 h light/day cycle at 19.1–22.7°C with relative humidity (RH) of 39–65%. All the animals were caged with ready availability of food and water.

### 2.3. Toxicity Experiments

#### 2.3.1. Single Dose Acute Oral Toxicity Analysis

Three female rats with average age of 6-7 weeks were used for single dose acute oral toxicity analysis. In brief, AND-2-HyP-*β*-CYD complex [10] at the dose of 2000 mg/kg was administered to female rats through the oral route of administration according to OECD guideline 423 [13]. Animals were closely observed initially every 4 h, followed by once a day for a period of 14 days for any signs of toxicity or mortality, such as occurrence of lacrimation, changes in pupil size, and presence of an unusual respiratory pattern along with response to handling, as well as presence of clonic or tonic movements, stereotypes, or bizarre behaviour [14]. In addition, food and water consumption was recorded at alternate days. On the other hand, bodyweight was recorded weekly. At the end of the study on 14^th^ day, all the animals were observed for food intake, bodyweight, gross behavioral changes, and mortality. Animals were sacrificed after 14^th^ day of the experimental protocol using ketamine (0.35 mL/kg) and xylazine (0.10 mL/kg) intraperitoneally, and gross necropsy was done to notice any alteration such as change in size, color, and architecture of organs.

#### 2.3.2. Repeated Dose Subacute Oral Toxicity Analysis

Seventy-two rats (36 males and 36 females) with average age of 6-7 weeks were randomly selected and grouped into low dose (1/10 of LD50 dose, 200 mg/kg), medium dose (1/5 of LD50, 400 mg/kg), high dose (1/3 of LD50, 666 mg/kg), reversal control of high dose (666 mg/kg), reversal control, and normal control. Rats were administered once daily the solution of AND-2-HyP-*β*-CYD complex by oral gavage as per the schedule throughout the experiment for 28 consecutive days according to OECD guideline 407. All the animals were strictly observed for mortality and morbidity in addition to clinical signs for a period of 28 days, followed by 14 additional days for evaluating reversal effects. Additionally, food and water consumption was recorded at alternate days, whereas bodyweight was documented weekly.

In order to carry out the hematological and biochemical evaluation, blood samples were collected on 28^th^ and 42^nd^ day through retroorbital vein. Furthermore, animals were sacrificed for gross necropsy and subjected to fastidious evaluation of external body surface including all the orifices, cranial, thoracic, and abdominal cavities along with their contents. Following analysis of gross necropsy, the liver and kidney in addition to other organs were removed surgically, weighed, and stored at −40°C in 10% formalin solution. The liver and kidney were studied for further histopathological examination.

#### 2.3.3. Single Dose Acute Inhalation Toxicity Analysis

Thirty SD rats with average age of 6-7 weeks were randomly selected and grouped into 3 groups, and each group was constituted with 5 males and 5 females as per OECD guideline 403. The animals in the control group did not receive any vehicle or treatment, while citrate buffer (pH 6.5) was administered in the vehicle control group through nebulization [15] as liquid aerosols. The animals in the treatment group were exposed to AND-2-HyP-*β*-CYD complex in citrate buffer (pH 6.5) through nebulization at the dose of 5 mg/L for 4 h. All the animals were examined cautiously for any clinical signs related to gross behavioral changes and mortality along with the recording of food and water consumption at alternate days, whereas change in bodyweight was plotted weekly.

#### 2.3.4. Repeated Dose Subacute Inhalation Toxicity Analysis

Sixty SD rats with average age of 6-7 weeks were randomly selected and differentiated into 2 groups (30 males and 30 females). The two groups were further subdivided into the normal control group, vehicle control group (citrate buffer, pH 6.5), and low dose (1/10 of MTD dose, i.e., 0.5 mg/L/4 h), medium dose (1/5 of MTD dose, i.e., 1 mg/L/4 h), and high dose (1/3 of MTD, i.e., 1.66 mg/L/4 h) groups. Rats were exposed once daily AND-2-HyP-*β*-CYD solution by nebulization as per the schedule throughout the experiment for 28 consecutive days according to OECD guideline 412. All the animals were observed for mortality and morbidity in addition to clinical signs for a period of 28 days in addition to recording of food and water consumption at alternate days, whereas bodyweight was assessed weekly.

To conduct the hematological and biochemical evaluation, blood samples were collected on 28^th^ day through retroorbital vein. In addition, animals were sacrificed after blood collection and subjected to gross necropsy including a careful examination of the external surface. Following analysis of gross necropsy, the liver, kidney, and lungs in addition to other organs were removed surgically, weighed, and stored at –40°C in 10% formalin solution. The liver, kidney, and lungs tissues were studied for further histopathological examination.

### 2.4. Hematological Analysis

Blood samples collected in heparinized tubes were examined using an automated hematology system at a commercial diagnostic laboratory. The blood samples were evaluated for leukocytes (WBC), erythrocytes (RBC), haemoglobin (Hb), haematocrit (HCT), mean corpuscular volume (MCV), mean corpuscular haemoglobin (MCH), mean corpuscular haemoglobin concentration (MCHC), platelet count (PLT), neutrophils (NEUT), monocytes (MONO), eosinophils (EOS), and basophils (BASO).

### 2.5. Serum Biochemical Analysis

To obtain samples for serum analysis, blood samples were collected into sterile tubes without any anticoagulant coating and allowed to stand for 30 min. The samples were centrifuged at 1500 g for 10 min at 4°C. The supernatant was collected and stored at 4°C till further processing. Serum samples were analyzed for albumin (ALB), alkaline phosphatase (ALP), alanine aminotransferase (ALT), aspartate aminotransferase (AST), bilirubin (BIL), calcium (CA), cholesterol (CHO), creatinine (CREJ), phosphorous (PHOS), total protein (TP), urea, and glucose (GLU) level by using standard diagnostic test kits on a semiautomated clinical biochemistry analyzer at a commercial laboratory.

### 2.6. Histopathological Assessment

The organs collected for histopathology analysis were embedded in paraffin wax, sectioned with microtome, and stained by hematoxylin and eosin (H&E) dye. Blinded histological analysis was performed by a trained pathologist as per the score of 0, none; 1, mild; 2, moderate; and 3, severe.

### 2.7. Statistical Analysis

Data obtained for various studies were expressed as mean value along with standard deviation (mean ± SD). All the statistical analysis were performed by one-way analysis of variance (ANOVA) followed by Dunnett's test, and the graphs were drawn by using GraphPad Prism 5.0 (GraphPad Prism, San Diego, USA), and *p* value <0.05 was considered statistically significant.

## 3. Results and Discussion

### 3.1. Acute and Subacute Oral Toxicity Analysis of AND-2-HyP-*β*-CYD Complex

A large population in developing countries depends on phytomolecules-based formulations [16–18] for their treatment. However, very limited scientific literature is available regarding the safety and efficacy of traditional medicines [19]. This necessitates to carry out the toxicological studies to serve two purposes, i.e., establishment of dose ranges in preclinical studies and disseminating the data on the safety profile of phytomolecules prior to product development [20]. The first sign of toxicity over repeated exposure of any substance is aberrant alteration in body and organ weights; and therefore, they are considered as vital indicators for adverse effects [21].

The single dose acute oral toxicity analysis at 2000 mg/kg of AND-2-HyP-*β*-CYD complex indicated that all the animals were in somnolence condition with decreased motor activity. No abnormality was detected in gross pathology of rats (Supplementary Table 1). Hence, LD50 (lethal dose) was found to be >2000 mg/kg as per the OECD 423 guidelines. Apart from this, there was no remarkable fluctuation in the bodyweights of the treated animal group as compared to the control group after the 28-day treatment period in subacute oral toxicity analysis (Supplementary Table 2). The data collected for food-water intake were found to be normal and weight gain showed gradual increase during the study, thereby inferring the nontoxic effect of the AND-2-HyP-*β*-CYD complex on the growth of the animals. Moreover, there was no remarkable difference in the organs weight of control and treatment groups in subacute oral toxicity analysis (Supplementary Table 3). Organ weight indicates the pathological and physiological status of animals, and it is a beneficial parameter in toxicity studies as it plays an important role in toxicity prediction, enzyme induction, physiologic perturbations, and acute injury; correlation to any histopathological changes; and little interanimal variability [22].

The hematopoietic system is one of the highly sensitive sites for toxicity and is a vital indicator of the pathological and physiological conditions in humans and animals [23]. Marginal fluctuations in hematological parameters provide greater prognostic factors for drug-induced toxicity [24]. Likewise, oral consumption of AND-2-HyP-*β*-CYD complex had no undesirable consequences on the circulating blood cells as well as on their production (Table 1), except significant increment in PLT level (×10^3^ cells/*μ*L) in low dose (1062.33 ± 38.75), reversal control (1316.83 ± 61.02), and reversal control of high dose (1246.67 ± 42.52) in comparison to the control group (928.67 ± 0.34). Correspondingly, similar results were also noticed in female rats (Table 1). Previous reports indicated that AND augments the PLT count owing to its broad-spectrum antiviral activity [25, 26]. Furthermore, a significant increase in EOS (%) was noticed in the reversal control group (1.15 ± 0.26) in female rats as compared to the control group (0.42 ± 0.10). This may be attributed to minor allergic reactions [27] in the reversal control group as it is not observed in other treatment groups of male and female rats.

**Figure 1:**
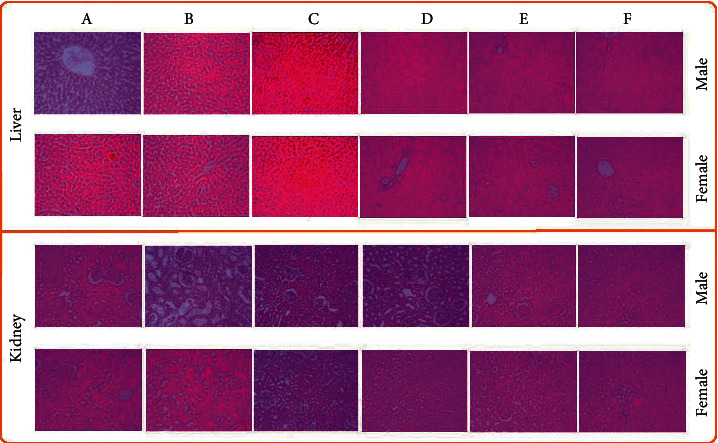
Photomicrographs of the liver and kidney of male and female rats in subacute oral toxicity of AND-2-HyP-*β*-CYD complex: (a) normal control, (b) low dose (1/10 of LD50 dose, 200 mg/kg), (c) medium dose (1/5 of LD50, 400 mg/kg), (d) high dose (1/3 of LD50, 666 mg/kg), (e) reversal control of high dose (666 mg/kg), and (f) reversal control. Magnification of 40x was used. No observable signs of toxicity are found in any doses and groups.

Following this, biochemical parameters were also estimated in AND-2-HyP-*β*-CYD complex treatment groups in subacute oral toxicity analysis in both male and female rats (Table 2). AND-2-HyP-*β*-CYD complex significantly augmented the AST (U/L) level in moderate dose (127.83 ± 10.22), high dose (131.0 ± 6.50), and reversal control of high dose (129.60 ± 16.10) in comparison to the control group (90.17 ± 9.92) in female rats. This was coinciding with the previous report [28]. Subsequently, histopathological analysis was carried out under subacute oral toxicity analysis for the liver and kidney as shown in Figure 1 in both male and female rats. Photomicrographs of histopathology of the liver of male and female rats indicated inflammatory changes with overall unremarkable lesion score of +1 [29, 30] (Figure 1). Moreover, no degenerative and necrotic changes were observed in all treated and normal groups of male rats. This may be correlated with the hematological and biochemical parameters estimated (Tables 1 and 2). Hence, it may be speculated that augmented AST level, PLT level, and EOS (%) may be attributed to minor inflammatory score of +1 [31]. In addition, histopathology of the kidney of male and female rats treated with AND-2-HyP-*β*-CYD complex through the oral route of administration did not exhibit any alterations in terms of vascular, degenerative, and necrotic changes of renal tubules (Figure 1). Hence, male and female rats treated with oral doses of 200, 400, and 666 mg/kg of AND-2-HyP-*β*-CYD complex did not exhibit any noteworthy signs of abnormalities. The NOAEL (no observed adverse effect level) was found to be 666 mg/kg for AND-2-HyP-*β*-CYD complex.

### 3.2. Acute and Subacute Inhalation Toxicity Analysis of AND-2-HyP-*β*-CYD Complex

The single dose (5 mg/L/4 h) acute inhalation toxicity analysis of AND-2-HyP-*β*-CYD complex in SD rats indicates no abnormality in the 14-day study. Hence, MTD (maximum tolerated dose) of AND-2-HyP-*β*-CYD complex was found to be >5 mg/L/4 h. Following this, the subacute inhalation toxicity study (28 days) was conducted in SD rats with the normal control group, vehicle control group (citrate buffer, pH 6.5), and low dose (1/10 of MTD dose, i.e., 0.5 mg/L/4 h), medium dose (1/5 of MTD dose, i.e., 1 mg/L/4 h), and high dose (1/3 of MTD, i.e., 1.66 mg/L/4 h) of AND-2-HyP-*β*-CYD complex. The bodyweight gain and organ weight were found to be normal in all groups of male and female rats treated with AND-2-HyP-*β*-CYD complex through nebulization (Supplementary Table 4 and Supplementary Table 5). Consumption of AND-2-HyP-*β*-CYD complex via the inhalation route of administration slightly increased the RBC (×10^6^ cells/*μ*L) level in all treated groups (Table 3) in both male and female rats with no significant difference. This effect may be attributed to the presence of sodium citrate as excipient in citrate buffer in addition to variation in the normal range of RBCs according to age [32].  Table 4 provides the data of biochemical parameters in both male and female rats treated with AND-2-HyP-*β*-CYD complex via the inhalation route of administration. There was no significant difference in biochemical parameters estimated in male and female rats in comparison to the control group (Table 4) except significant decrease in GLU (mg/dL) level at high dose of 1.66 mg/L/4 h in female rats. The low GLU level may be coincided with higher indexes of inflammation and oxidative stress in healthy subject [33].

**Figure 2:**
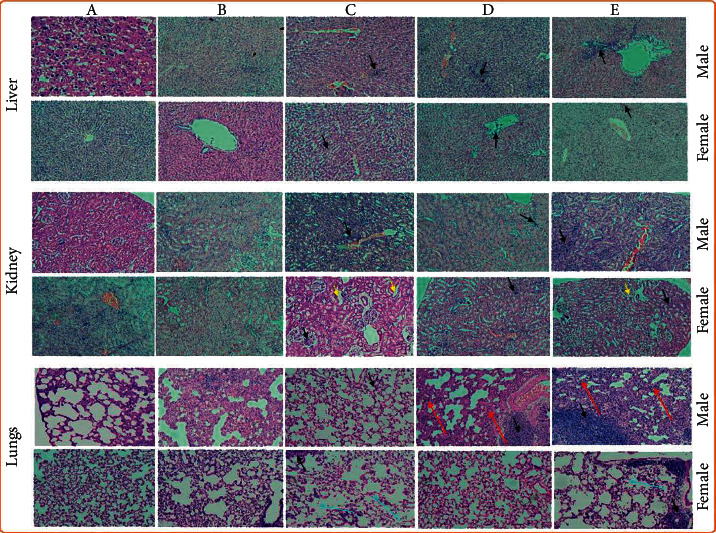
Photomicrographs of the liver, kidney and lungs of male and female rats in subacute inhalation toxicity of AND-2-HyP-*β*-CYD complex: (a) normal control, (b) vehicle control (citrate buffer, pH 6.5), (c) low dose (0.5 mg/L/4 h), (d) medium dose (1 mg/L/4 h), and (e) high dose (1.66 mg/L/4 h). Magnification of 40x was used. Black arrow indicates the presence of inflammation in the lungs, liver, and kidney of both the sexes at low, medium, and high doses. Yellow arrow denotes the degenerative changes in the kidney of medium and highest doses. Red arrows in the lungs of male rats at mid and high doses show sign of decongestion, whereas blue arrows in the lungs of female rats at low and high doses reveal degenerative changes in perialveolar tissues.

The histopathological photomicrographs for the subacute inhalation toxicity study are shown in Figure 2. Photomicrographs indicated overall lesion score of +3 in the liver at high dose of 1.66 mg/L/4 h in comparison to +1 in the liver of normal male and female rats. On the other hand, mid dose (1 mg/L/4 h) and low dose (0.5 mg/L/4 h) exhibited the overall lesion score of +2 in the liver of male and female rats with necrosis in hepatocytes (Figure 2). These changes could not be very well correlated with AST, ALT, and bilirubin levels. Based on the parameters observed in hematology and biochemistry, none of the groups showed any significant variation in liver markers (Table 4). The previous study also indicated that AND did not induce any toxicity at 500 mg/kg dose [34]. Hence, we may assume these changes might be due to some other biological (genetic or epigenetics) variations [35] or the vehicle effect or any oxidative stress [36]. Correspondingly, identical results were also obtained in the kidney and lungs tissues of both male and female rats with overall lesion score of +3 in high dose, +2 in mid dose, and +1 in the vehicle control group in addition to necrotic alterations in the kidney and emphysema in alveoli of the lungs. Emphysema refers to damage to the walls of the alveoli of the lungs (Figure 2). VEGF (vascular endothelial growth factor) acts on a large number of lung tissue cells, including alveolar type II cells and vascular smooth muscle cells. Emphysema usually develops as a consequence of treatment with a VEGF receptor-targeting drug [37]. Hence, we suppose that AND being an antiangiogenic drug might have bind to the VEGF receptor [38] and consequently promoted the emphysema in lung tissue in dose-dependent manner. Hence, AND-2-HyP-*β*-CYD complex via the inhalation route of administration exhibited mild to moderate toxicity at higher dose. Based on the results obtained from biochemical, hematological, and histopathological analyses, the NOAEL was found to be 1/10 of MTD (0.5 mg/L/4 h/day) and LOAEL was found to be 1/5 of MTD (1 mg/L/4 h/day). Hence, the findings of the present study would further be useful in assessing and utilizing the medicinal and therapeutic benefits of AND-2-HyP-*β*-CYD complex.

## 4. Conclusion

In conclusion, single dose oral administration of AND-2-HyP-*β*-CYD complex at 2000 mg/kg indicated no abnormality in gross pathology of rats. In addition, hematological, biochemical, and histopathological analysis after subacute toxicity (200, 400, and 666 mg/kg of AND-2-HyP-*β*-CYD) study did not exhibit any noteworthy signs of abnormalities. On the other hand, single dose (5 mg/L/4 h) acute inhalation toxicity analyses of AND-2-HyP-*β*-CYD complex indicated MTD of >5 mg/L/4 h. Subacute inhalation toxicity of AND-2-HyP-*β*-CYD complex exhibited significant toxicity at higher dose, eventhough it could not be well correlated with the hematological and biochemical parameters. Hence, the NOAEL was found to be 1/10 of MTD (0.5 mg/L/4 h/day) and LOAEL was noticed to be 1/5 of MTD (1 mg/L/4 h/day). The results of acute and subacute toxicity analysis of AND-2-HyP-*β*-CYD complex provide valuable preliminary data on the toxic profile. However, further assessments such as genotoxicity and reproductive toxicity are required to proceed for clinical studies of AND-2-HyP-*β*-CYD complex. Eventually, it is mandatory to understand that phytomolecules should be analyzed under a set of stringent parameters for translating into a clinically viable product.

## Figures and Tables

**Table 1:** Evaluation of hematological parameters in repeated dose oral subacute toxicity study of AND-2-HyP-*β*-CYD complex.

Groups	Parameters	Normal control	Low dose (200 mg/kg)	Moderate dose (400 mg/kg)	High dose (666 mg/kg)	Reversal control	Reversal control of high dose (666 mg/kg)
Male	WBCB (×10^3^ cells/*μ*L)	2.36 ± 0.09	3.17 ± 0.29	2.40 ± 0.22	2.32 ± 0.26	1.53 ± 0.17	2.09 ± 0.14
	RBC (×10^6^ cells/*μ*L	5.57 ± 0.09	5.43 ± 0.05	6.39 ± 0.31	7.00 ± 0.04	5.24 ± 0.40	6.93 ± 0.32
	Mean HGB (g/dL)	12.88 ± 0.49	12.28 ± 0.51	12.70 ± 0.78	12.48 ± 0.59	9.25 ± 0.47	12.63 ± 0.59
	HCT (%)	39.73 ± 1.7	39.83 ± 0.75	38.77 ± 1.97	39.93 ± 1.23	37.67 ± 1.41	39.00 ± 0.87
	MCV (fL)	51.80 ± 0.45	54.83 ± 0.74	52.32 ± 1.49	52.48 ± 0.61	57.42 ± 2.38	54.80 ± 1.24
	MCH (pg)	15.07 ± 0.22	16.13 ± 0.34	14.58 ± 0.44	14.00 ± 0.73	14.63 ± 0.54	14.47 ± 0.86
	MCHC (g/dL)	28.95 ± 0.27	29.83 ± 0.59	30.50 ± 1.26	31.33 ± 0.67	32.03 ± 1.14	32.33 ± 0.99
	PLT (×10^3^ cells/*μ*L)	928.67 ± 0.34	^*∗*^1062.33 ± 38.75	1042.83 ± 78.54	991.00 ± 54.04	^*∗*^1316.83 ± 61.02	^*∗*^1246.67 ± 42.52
	NEUT (%)	11.56 ± 1.34	19.62 ± 1.96	17.92 ± 2.59	12.95 ± 0.84	18.00 ± 2.14	16.50 ± 2.30
	LYM (%)	84.55 ± 1.74	76.72 ± 3.52	69.00 ± 7.06	81.03 ± 1.57	72.48 ± 3.20	68.92 ± 13.04
	MONO (%)	3.57 ± 0.29	3.08 ± 0.14	3.38 ± 0.33	3.62 ± 0.24	3.95 ± 0.69	4.20 ± 0.62
	EOS (%)^*∗*^	0.61 ± 0.10	0.43 ± 0.07	0.50 ± 0.07	0.65 ± 0.08	0.65 ± 0.09	1.08 ± 0.11
	BASO (%)	0.57 ± 0.13	0.65 ± 0.14	0.38 ± 0.12	0.45 ± 0.10	0.42 ± 0.14	0.63 ± 0.09

Female	WBCB (×10^3^ cells/*μ*L)	2.51 ± 0.25	3.73 ± 0.35	1.96 ± 0.42	2.18 ± 0.30	1.53 ± 0.17	1.83 ± 0.15
	RBC (×10^6^ cells/*μ*L	5.68 ± 0.16	5.47 ± 0.36	6.53 ± 0.13	7.18 ± 0.31	5.24 ± 0.40	5.97 ± 0.20
	Mean HGB (g/dL)	10.35 ± 0.40	9.72 ± 0.32	9.87 ± 0.37	9.65 ± 0.24	9.25 ± 0.47	8.77 ± 0.27
	HCT (%)	43.33 ± 2.14	43.67 ± 1.80	39.00 ± 1.65	42.27 ± 1.49	37.67 ± 1.41	45.33 ± 1.71
	MCV (fL)	52.92 ± 0.67	54.75 ± 1.19	51.97 ± 0.31	50.72 ± 0.65	57.42 ± 2.38	56.15 ± 0.88
	MCH (pg)	15.02 ± 0.37	16.43 ± 1.37	15.65 ± 0.88	15.00 ± 0.52	14.63 ± 0.54	14.88 ± 0.80
	MCHC (g/dL)	28.72 ± 0.74	32.18 ± 2.45	31.67 ± 1.71	32.83 ± 0.65	32.03 ± 1.14	33.97 ± 1.48
	^*∗*^PLT (×10^3^ cells/*μ*L)	927.00 ± 74.40	1127.67 ± 70.26	1191.00 ± 39.98	1286.33 ± 77.03	1316.83 ± 61.02	1130.00 ± 53.42
	NEUT (%)	15.55 ± 1.76	23.80 ± 4.05	17.92 ± 2.36	15.68 ± 1.59	18.00 ± 2.14	15.00 ± 2.21
	LYM (%)	77.30 ± 2.29	61.28 ± 6.58	70.70 ± 4.08	76.80 ± 2.43	72.48 ± 3.20	77.22 ± 3.61
	MONO (%)	3.32 ± 0.39	3.33 ± 0.36	3.93 ± 0.32	4.25 ± 0.90	3.95 ± 0.69	3.45 ± 0.35
	EOS (%)	0.42 ± 0.10	0.53 ± 0.11	0.65 ± 0.06	0.58 ± 0.09	^*∗*^1.15 ± 0.26	0.70 ± 0.07
	BASO (%)	0.58 ± 0.12	0.55 ± 0.10	0.52 ± 0.08	0.42 ± 0.12	0.42 ± 0.14	0.60 ± 0.10

Each value represents the mean ± standard deviation (*n* = 6). One-way ANOVA test (*P* > 0.05) followed by Dunnett's test. ^*∗*^(*P* < 0.05) significantly different.

**Table 2:** Evaluation of biochemical parameters in the repeated oral dose subacute toxicity study of AND-2-HyP-*β*-CYD complex.

Groups	Parameters	Normal control	Low dose (200 mg/kg)	Moderate dose (400 mg/kg)	High dose (666 mg/kg)	Reversal control	Reversal control of high dose (666 mg/kg)
Male	ALB (g/dL)	3.84 ± 0.19	3.40 ± 0.36	3.81 ± 0.39	3.99 ± 0.14	3.72 ± 0.16	3.70 ± 0.19
	ALP (IU/L)	121.0 ± 15.13	106.33 ± 3.45	102.83 ± 4.96	104.67 ± 4.39	106.67 ± 4.27	107.83 ± 7.04
	ALT (U/L)	37.62 ± 4.03	40.67 ± 3.96	33.50 ± 3.07	41.83 ± 2.88	39.67 ± 3.40	44.67 ± 4.36
	AST (U/L)	91.50 ± 3.71	99.67 ± 8.52	109.17 ± 17.51	133.0 ± 6.78	109.0 ± 10.73	109.33 ± 9.01
	BIL (U/L)	0.10 ± 0.00	0.10 ± 0.00	0.10 ± 0.00	0.10 ± 0.00	0.10 ± 0.00	0.10 ± 0.00
	CA (mg/dL)	9.80 ± 0.33	8.02 ± 1.37	9.52 ± 0.23	10.28 ± 0.18	9.33 ± 0.15	9.37 ± 0.37
	CHO (mg/dL)	46.0 ± 4.23	42.83 ± 3.75	46.67 ± 2.82	43.17 ± 2.24	49.83 ± 2.33	46.00 ± 4.07
	CREJ (mg/dL)	0.26 ± 0.02	0.26 ± 0.02	0.35 ± 0.03	0.28 ± 0.03	0.38 ± 0.03	0.32 ± 0.03
	PHOS (mg/dL)	7.0 ± 0.56	7.65 ± 0.56	7.47 ± 0.57	8.53 ± 0.59	7.97 ± 0.50	8.00 ± 0.62
	TP (g/dL)	6.25 ± 0.30	6.72 ± 0.21	6.28 ± 0.26	6.02 ± 0.29	6.52 ± 0.25	6.05 ± 0.24
	UREA (mg/dL)	18.17 ± 0.48	17.0 ± 1.03	15.83 ± 1.11	16.67 ± 0.80	16.00 ± 0.82	16.50 ± 1.34
	GLU (mg/dL)	128.33 ± 3.69	128.17 ± 4.83	122.67 ± 3.90	129.0 ± 3.50	129.50 ± 4.49	141.67 ± 5.08

Female	ALB (g/dL)	4.02 ± 0.23	3.87 ± 0.31	3.94 ± 0.22	3.87 ± 0.27	3.92 ± 0.16	3.73 ± 0.26
	ALP (U/L)	98.50 ± 5.19	100.67 ± 6.29	103.0 ± 6.29	100.83 ± 6.37	97.0 ± 6.69	153.40 ± 17.38^*∗*^
	ALT (U/L)	43.50 ± 3.89	45.83 ± 2.57	39.33 ± 3.94	38.0 ± 3.69	43.33 ± 4.39	41.83 ± 3.85
	AST (U/L)	90.17 ± 9.92	93.0 ± 3.79	^*∗*^127.83 ± 10.22	^*∗*^131.0 ± 6.50	98.17 ± 10.46	^*∗*^129.60 ± 16.10
	BIL (U/L)	0.10 ± 0.00	0.10 ± 0.00	0.10 ± 0.00	0.10 ± 0.00	0.10 ± 0.00	0.10 ± 0.00
	CA (mg/dL)	8.85 ± 0.37	9.67 ± 0.17	9.72 ± 0.10	9.45 ± 0.27	8.79 ± 0.31	8.72 ± 0.20
	CHO (mg/dL)	43.0 ± 2.68	54.33 ± 3.73	43.17 ± 2.86	50.83 ± 2.21	48.0 ± 3.09	41.60 ± 1.97
	CREJ (mg/dL)	0.30 ± 0.03	0.30 ± 0.03	0.39 ± 0.04	0.31 ± 0.03	0.21 ± 0.04	0.31 ± 0.03
	PHOS (mg/dL)	8.55 ± 0.39	7.83 ± 0.54	7.73 ± 0.72	7.18 ± 0.46	8.05 ± 0.43	8.06 ± 0.80
	TP (g/dL)	6.80 ± 0.16	6.03 ± 0.27	6.25 ± 0.35	6.37 ± 0.25	5.90 ± 0.32	5.86 ± 0.26
	UREA (mg/dL)	16.50 ± 0.96	16.50 ± 0.99	15.0 ± 0.89	19.67 ± 1.09	17.33 ± 0.99	23.0 ± 1.04
	GLU (mg/dL)	130.83 ± 6.26	118.17 ± 4.36	114.17 ± 5.49	134.33 ± 7.74	136.50 ± 4.33	129.0 ± 5.02

Each value represents the mean ± standard deviation (*n* = 6). One-way ANOVA test (*P* > 0.05) followed by Dunnett's test. ^*∗*^*P* < 0.05, one-way ANOVA test with Dunnett's test.

**Table 3:** Evaluation of hematological parameters in the inhalation subacute toxicity study of AND-2-HyP-*β*-CYD complex.

Groups	Parameters	Normal control	Vehicle control	Low dose	Mid dose	High dose
Male	WBCB (×10^3^ cells/*μ*L)	6.18 ± 3.03	7.41 ± 4.24	13.32 ± 2.54	7.44 ± 3.2	8.19 ± 4.47
	RBC (×10^6^ cells/*μ*L)	5.80 ± 2.16	8.76 ± 1.94	8.24 ± 0.41	8.19 ± 0.63	8.16 ± 1.17
	Mean HGB (g/dL)	11.67 ± 3.44	15.67 ± 2.73	14.8 ± 0.85	15.05 ± 0.45	15.1 ± 1.40
	HCT (%)	47.5 ± 10.47	52.75 ± 10.21	47.6 ± 2.19	49.75 ± 1.7	48 ± 7.41
	MCV (fL)	86.0 ± 14.49	61.0 ± 2.44	57.6 ± 1.51	61 ± 3.16	59 ± 2.91
	MCH (pg)	20.0 ± 1.82	17.75 ± 0.95	17.6 ± 0.54	18.25 ± 0.95	18.2 ± 1.09
	MCHC (g/dL)	23.5 ± 2.38	29.25 ± 1.25	30.8 ± 0.83	30 ± 0.00	31 ± 2.91
	PLT (×10^3^ cells/*μ*L)	637.75 ± 84.26	440.5 ± 122.56	568.2 ± 18.72	444.5 ± 109.36	466.6 ± 208.42
	NEUT (%)	16.75 ± 4.78	15.5 ± 8.06	15 ± 3.67	16.25 ± 4.5	15.2 ± 2.58
	LYM (%)	61 ± 10.78	69.5 ± 7.89	69.6 ± 9.28	64 ± 6.05	71.4 ± 1.81
	MONO (%)	15.5 ± 6.55	9.25 ± 4.57	7.8 ± 6.26	12.25 ± 6.13	7.4 ± 3.04
	EOS (%)	1.0 ± 0.00	1.75 ± 0.95	1.6 ± 0.54	2.75 ± 0.95	1.2 ± 0.44
	BASO (%)	5.75 ± 1.70	4.0 ± 1.15	6.0 ± 1.87	4.75 ± 1.25	5.2 ± 1.78

Female	WBCB (×10^3^ cells/*μ*L)	5.74 ± 1.83	7.43 ± 1.00	6.82 ± 1.26	6.64 ± 2.10	6.57 ± 1.20
	RBC (×10^6^ cells/*μ*L)	8.0 ± 0.44	7.91 ± 0.20	8.0 ± 1.59	8.1 ± 0.42	8.2 ± 0.37
	Mean HGB (g/dL)	14.35 ± 0.75	14.4 ± 0.47	14.88 ± 2.3	14.44 ± 0.75	14.58 ± 0.78
	HCT (%)	49.0 ± 3.57	49.66 ± 1.63	47.83 ± 9.57	47.8 ± 2.16	48.6 ± 2.50
	MCV (fL)	60.83 ± 1.83	62.66 ± 1.03	59.66 ± 2.73	59.0 ± 2.00	58.8 ± 0.83
	MCH (pg)	17.5 ± 0.54	17.83 ± 0.40	18.5 ± 1.22	17.4 ± 0.54	17.0 ± 0.00
	MCHC (g/dL)	28.66 ± 0.51	28.0 ± 0.00	31.0 ± 2.0	29.8 ± 0.44	29.2 ± 0.44
	PLT (×10^3^ cells/*μ*L)	679 ± 125.95	767.16 ± 49.83	684.16 ± 71.19	768.8 ± 109.26	822.4 ± 231.08
	NEUT (%)	17.16 ± 2.85	21.0 ± 5.25	17.33 ± 6.91	12.6 ± 8.29	17.6 ± 5.12
	LYM (%)	67.0 ± 6.606	66.0 ± 7.29	72.66 ± 8.64	73.4 ± 9.23	71.0 ± 4.47
	MONO (%)	8.16 ± 3.81	7.0 ± 2.52	7.0 ± 2.60	10.2 ± 0.54	7.4 ± 4.15
	EOS (%)	1.5 ± 0.54	1.0 ± 0.00	1.0 ± 0.00	1.6 ± 0.54	1.0 ± 0.00
	BASO (%)	6.33 ± 2.5	5.16 ± 1.72	2.0 ± 0.63	2.2 ± 0.83	3.0 ± 0.70

Each value represents the mean ± standard deviation (*n* = 6). One-way ANOVA test (*P* > 0.05) followed by Dunnett's test.

**Table 4:** Evaluation of biochemical parameters in the repeated dose inhalation subacute toxicity study of AND-2-HyP-*β*-CYD complex.

Groups	Parameters	Normal control	Vehicle control	Low dose	Mid dose	High dose
Male	ALB (g/dL)	4.6 ± 0.82	3.4 ± 0.25	3.16 ± 0.16	3.2 ± 0.33	3.3 ± 0.33
	ALP (U/L)	265.25 ± 88.48	305 ± 28.49	349.4 ± 90.54	348.0 ± 57.69	347.0 ± 137.17
	ALT (U/L)	38.75 ± 28.49	41.25 ± 14.93	32.0 ± 50.48	42.40 ± 12.40	42.0 ± 11.51
	AST (U/L)	88.75 ± 17.5	90 ± 30.27	81.0 ± 36.97	90.0 ± 11.18	91.0 ± 27.47
	BIL (U/L)	0.10 ± 0.00	0.10 ± 0.00	0.10 ± 0.00	0.10 ± 0.00	0.10 ± 0.00
	CA (mg/dL)	9.2 ± 0.52	9.25 ± 0.34	8.68 ± 0.63	8.78 ± 0.35	8.44 ± 0.30
	CHO (mg/dL)	47.0 ± 2.30	51.25 ± 7.22	46.8 ± 2.68	46.2 ± 2.16	48.6 ± 5.36
	CREJ (mg/dL)	0.3 ± 0.00	0.32 ± 0.05	0.32 ± 0.04	0.26 ± 0.05	0.32 ± 0.04
	PHOS (mg/dL)	9.95 ± 1.39	9.5 ± 3.0	14.62 ± 3.19	11.64 ± 1.17	12.02 ± 0.91
	TP (g/dL)	8.12 ± 0.77	7.2 ± 0.25	7.18 ± 0.30	6.9 ± 0.23	7.1 ± 0.23
	UREA (mg/dL)	33.85 ± 1.27	29.97 ± 3.79	34.62 ± 4.74	38.34 ± 2.57	34.08 ± 2.66
	GLU (mg/dL)	99.5 ± 8.22	99.5 ± 3.69	106.8 ± 9.44	103.4 ± 7.40	105.2 ± 11.32

Female	ALB (g/dL)	3.8 ± 0.38	3.68 ± 0.24	3.26 ± 0.19	3.18 ± 0.30	3.4 ± 0.6
	ALP (U/L)	205.16 ± 35.0	213.66 ± 53.26	241.66 ± 47.94	223.2 ± 39.35	215.4 ± 23.64
	ALT (U/L)	23.33 ± 4.08	27.5 ± 7.58	40.0 ± 19.23	40.0 ± 16.58	35.0 ± 16.58
	AST (U/L)	54.16 ± 14.97	66.66 ± 22.73	134.33 ± 55.16	116.4 ± 54.73	121.8 ± 63.51
	BIL (U/L)	0.10 ± 0.00	0.10 ± 0.00	0.10 ± 0.00	0.10 ± 0.00	0.10 ± 0.00
	CA (mg/dL)	9.88 ± 0.31	9.26 ± 0.35	8.71 ± 0.18	9.92 ± 0.59	9.14 ± 0.31
	CHO (mg/dL)	68.33 ± 9.39	56.5 ± 7.34	52.5 ± 5.92	54.2 ± 5.63	54.6 ± 9.44
	CREJ (mg/dL)	0.35 ± 0.04	0.38 ± 0.04	0.33 ± 0.05	0.36 ± 0.05	0.36 ± 0.05
	PHOS (mg/dL)	5.56 ± 0.47	6.68 ± 0.76	7.81 ± 0.87	6.88 ± 1.14	6.58 ± 0.74
	TP (g/dL)	7.4 ± 0.22	7.38 ± 0.29	7.31 ± 0.40	7.16 ± 0.27	7.28 ± 0.57
	UREA (mg/dL)	30.96 ± 3.64	32.38 ± 1.57	29.26 ± 1.09	30.14 ± 2.35	30.74 ± 3.64
	GLU (mg/dL)	115.16 ± 6.79	112 ± 5.32	113.66 ± 5.12	105.2 ± 9.36	^*∗*^91.4 ± 6.54

Each value represents the mean ± standard deviation (*n* = 6). One-way ANOVA test (*P* > 0.05) followed by Dunnett's test. ^*∗*^One-way ANOVA test (*P* < 0.05) followed by Dunnett's test.

## Data Availability

The data used to support the findings of this study are available from the corresponding author upon request.

## Supplementary Materials

Supplementary Materials Supplementary Table 1. Single dose acute oral toxicity study of AND-2-HyP-*β*-CYD complex. Supplementary Table 2. Measurement of bodyweight (g) in repeated dose oral toxicity analysis of AND-2-HyP-*β*-CYD complex. Supplementary Table 3. Measurement of organ weight (g) in repeated dose oral toxicity analysis of AND-2-HyP-*β*-CYD complex. Supplementary Table 4. Measurement of bodyweight (g) in repeated dose inhalation toxicity analysis of AND-2-HyP-*β*-CYD complex. Supplementary Table 5. Measurement of organ weight in repeated dose inhalation toxicity analysis of AND-2-HyP-*β*-CYD complex.
