# Supplementary material for: Acute and Subacute Toxicity Assessment of Andrographolide-2-hydroxypropyl-β-cyclodextrin Complex via Oral and Inhalation Route of Administration in Sprague-Dawley Rats
Source: ScientificWorldJournal. 2022 Mar 28;2022:6224107. doi: 10.1155/2022/6224107 (PMC8979680; doi:10.1155/2022/6224107)
Supplement: Supplementary Materials — Supplementary Table 1. Single dose acute oral toxicity study of AND-2-HyP-β-CYD complex. Supplementary Table 2. Measurement of bodyweight (g) in repeated dose oral toxicity analysis of AND-2-HyP-β-CYD complex. Supplementary Table 3. Measurement of organ weight (g) in repeated dose oral toxicity analysis of AND-2-HyP-β-CYD complex. Supplementary Table 4. Measurement of bodyweight (g) in repeated dose inhalation toxicity analysis of AND-2-HyP-β-CYD complex. Supplementary Table 5. Measurement of organ weight in repeated dose inhalation toxicity analysis of AND-2-HyP-β-CYD complex. [file 6224107.f1.doc]

**Suppl. Table 1**: Single dose acute oral toxicity study of AND-2-HyP-*β*-CYD complex

Animal Day Body weight (g) Food consumption (g) Clinical signs Mortality & Morbidity Gross Pathology

and

Weight gain (+g)

1. 0 169.8 - NAD - -

7 179.4 - NAD - -

14 191 (+21.2) 133.2 NAD NO NAD

2. 0 159.8 - NAD - -

1. 171.4 - NAD - -

14 184.5 (+24.7) 125.9 NAD NO NAD

3. 0 154.2 - NAD - -

7 166.8 - NAD - -

14 174.4 (+20.2) 126.9 NAD NO -

Note: NAD: No Abnormality Detected

**Suppl. Table 2: Measurement of body weight (g) in repeated dose oral toxicity analysis of AND-2-HyP-*β*-CYD complex**

Day Normal control Low dose Mid Dose High dose Reversal control Reversal control

of high dose

*Male ^#^Female Male Female Male Female Male Female Male Female Male Female

0 154.08±15.03 149.73±11.29 156.93±14.37 153.21±9.21 170.16±21.63 151.83±8.80 168.83±14.17 150.2±10.21 164.71±9.00 151.35±10.77 171.73±13.21 156.08±8.93

7 177.23±26.03 167.86±6.85 179.93±20.80 170.48±9.34 188.23±24.91 168.01±10.66 186.40±12.24 166.13±10.85 183.28±9.11 171.20±10.80 186.86±12.77 172.28±10.55

14 195.06±24.42 184.61±9.05 199.75±20.03 184.95±8.34 205.46±24.84 185.03±11.57 204.38±12.57 185.35±11.24 220.05±34.30 185.70±9.88 212.01±20.35 189.28±16.63

21 215.06±20.62 201.55±13.80 218.08±19.83 197.98±9.69 209.25±59.40 184.21±41.60 227.46±12.50 204.88±11.87 233.81±24.26 206.65±12.81 214.21±55.26 210.3±13.26

28 228.26±18.61 219.75±11.99 230.43±15.77 220.30±6.34 237.00±31.64 221.71±08.79 245.36±10.91 222.98±9.96 253.83±25.78 227.88±13.80 252.18±23.27 226.11±12.84

35 270.55±27.95 250.26±17.68 273.78±22.22 246.03±11.67

42 287.48±25.59 272.96±16.53 294.36±24.40 267.51±10.06

n=6, *(P>0.05, One-way ANOVA test followed by Duneett’s Multiple comparison test except reversal control (42 days) and reversal control of high dose (42 days) in male rats

n=6 ^#^(P>0.05, One-way ANOVA test followed by Duneett’s Multiple comparison test except reversal control (42 days) and reversal control of high dose (42 days) in female rats

**Suppl. Table 3: Measurement of organ weight (g) in repeated dose oral toxicity analysis of AND-2-HyP-*β*-CYD complex**

Group Heart Kidney Liver Spleen Lungs Brain Testes/Ovaries Pancreas

Right Left Right Left

Normal

Control

*Male 0.93±0.05 1.41±0.10 1.09±0.06 10.69±1.17 0.63±0.14 2.67±0.43 1.49±0.43 1.25±0.12 1.28±0.14 0.83±0.07

^#^Female 0.70±0.10 0.72±0.10 0.71±0.09 8.72±1.60 0.55±0.18 2.45±0.29 1.4±0.25 0.56±0.10 0.85±0.28

Low dose

Male 1.05±0.09 1.10±0.14 1.05±0.09 10.36±1.17 0.65±0.20 2.30±0.30 1.38±0.21 1.33±0.08 1.29±0.08 0.55±0.08

Female 0.82±0.13 0.75±0.09 0.72±0.08 8.64±0.95 0.71±0.23 2.28±0.45 1.48±0.21 0.90±0.49 0.65±0.15

Mid Dose

Male 0.91±0.11 1.05±0.12 1.05±0.10 9.77±1.12 0.66±0.14 2.34±0.20 1.6±0.20 1.37±0.10 1.37±0.12 0.57±0.11

Female 0.95±0.08 0.8±0.09 0.83±0.07 7.48±0.97 0.67±0.07 2.63±0.60 1.43±0.19 0.51±0.12 0.55±0.06

High Dose

Male 0.91±0.09 1.01±0.11 1.03±0.15 11.27±1.06 0.93±0.13 2.44±0.37 1.69±0.20 1.37±0.06 1.38±0.09 0.84±0.11

Female 0.76±0.08 0.74±0.07 0.72±0.07 8.44±1.42 0.98±0.61 1.84±0.23 1.45±0.22 0.55±0.23 0.82±0.17

Reversal

Control

Male 1.21±0.16 1.18±0.16 1.17±0.17 11.86±0.84 0.86±0.23 2.20±0.38 1.34±0.06 1.45±0.05 1.47±0.08 0.64±0.16

Female 0.68±0.18 0.80±0.14 0.80±0.16 8.94±1.21 0.72±0.19 2.02±0.24 1.49±0.32 0.63±0.15 0.60±0.24

Reversal of

High dose

Male 1.07±0.07 1.06±0.10 1.03±0.10 13.52±2.18 0.88±0.08 2.21±0.02 1.37±0.08 1.29±0.09 1.25±0.46 1.02±0.07

Female 0.71±0.08 0.77±0.08 0.76±0.10 8.25±0.83 0.70±0.06 2.21±0.57 1.32±0.15 0.76±0.10 0.88±0.09

n=6, *(P>0.05, One-way ANOVA test followed by Duneett’s Multiple comparison test except reversal control (42 days) and reversal control of high dose (42 days) in male rats

n=6 ^#^(P>0.05, One-way ANOVA test followed by Duneett’s Multiple comparison test except reversal control (42 days) and reversal control of high dose (42 days) in female rats

**Suppl. Table 4: Measurement of body weight (g) in repeated dose inhalation toxicity analysis of AND-2-HyP-*β*-CYD complex**

Day Normal control Vehicle Control Low Dose Mid dose High dose

Male Female Male Female Male Female Male Female Male Female

0 258.85±26.85 211.75±21.64 245.53±56.62 210.06±9.86 262.53±31.15 195.9±31.22 244.5±20.99 220.50±12.66 244.83±20.99 206.48±17.88

7 272.63±25.57 227.41±20.38 262.81±44.56 216.21±9.96 281.16±35.25 202.33±30.22 260.18±20.66 226.96±12.60 258.41±24.22 212.55±17.98

14 297.83±25.22 246.88±21.98 295.36±62.71 221.41±10.31 299.66±35.65 207.71±30.22 275.55±20.95 233.76±12.51 274.20±23.10 218.96±18.65

21 312.48±29.99 263.90±21.04 312.16±63.40 226.83±9.04 316.11±35.38 213.45±30.19 289.7±22.10 239.36±12.71 288.80±22.98 224.35±18.89

28 330.08±22.93 281.16±21.97 312.46±48.72 233.33±9.74 333.11±35.51 219.28±29.63 305.45±21.38 246.08±12.95 303.76±23.71 230.25±19.93

n=6, *(P>0.05, One-way ANOVA test followed by Duneett’s Multiple comparison test in male and female rats

**Suppl. Table 5: Measurement of organ weight in repeated dose inhalation toxicity analysis of AND-2-HyP-*β*-CYD complex**

Group Heart Kidney Liver Spleen Lungs Brain Testes/Ovaries Pancreas

Right Left Right Left

Normal Control

Male 1.15±011 1.14±0.010 1.08±0.08 10.19±0.61 0.97±0.08 3.09±0.12 1.36±0.10 1.52±0.11 1.52±0.08 1.69±0.08

Female 0.71±0.06 0.77±0.06 0.73±0.06 5.23±0.42 0.63±0.04 2.34±0.18 1.39±0.18 2.46±0.10 1.32±0.10

Vehicle control

Male 1.16±0.06 1.11±0.06 0.99±0.06 11.01±0.55 1.23±0.06 4.02±0.12 1.31±0.05 0.71±0.05 0.71±0.06 1.04±0.05

Female 0.79±0.11 0.80±0.11 0.68±0.05 2.02±0.08 0.67±0.05 2.71±0.11 1.31±0.04 1.14±0.1 1.03±0.05

Low Dose

Male 1.49±0.07 1.37±0.05 1.22±0.06 13.43±0.5 0.90±0.01 3.06±0.09 1.4±0.09 1.36±0.06 1.61±0.11 1.26±0.08

Female 0.82±0.06 0.72±0.05 0.71±0.03 7.00±0.07 0.60±0.06 3.00±0.09 1.37±0.05 0.70±0.013 0.99±0.04

Mid Dose

Male 1.68±0.08 1.26±0.16 1.17±0.03 13.2±1.98 1.5±0.09 2.1±0.06 1.13±0.06 0.83±0.01 0.76±0.01 1.36±0.04

Female 0.78±0.01 1.12±0.06 0.97±0.26 7.4±0.26 0.71±0.003 3.61±0.09 1.54±0.02 2.95±0.02 0.71±0.01

High dose

Male 1.17±0.09 1.03±0.04 0.9±0.01 10.64±0.01 1.20±0.10 2.90±0.09 1.27±0.07 0.92±0.05 0.9±0.01 0.91±0.01

Female 0.61±0.05 0.72±0.05 0.69±0.06 6.44±0.32 0.46±0.09 2.72±0.11 1.48±0.16 1.27±0.03 0.68±0.06

n=6, *(P>0.05, One-way ANOVA test followed by Duneett’s Multiple comparison test in male and female rats
